# Supplementary material for: A study on the promotion effect of government guidance on the construction of a national unified market logistics channel
Source: PLoS One. 2023 Nov 13;18(11):e0293969. doi: 10.1371/journal.pone.0293969 (PMC10642817; doi:10.1371/journal.pone.0293969)
Supplement: Attachment — Submitted filename: Response to Reviewers.docx [file pone.0293969.s001.docx]

**Dear editors and reviewers:**

We appreciate editors’ sincere letter and reviewers' helpful comments concerning our manuscript entitled “A Study on the Promotion Effect of Government Guidance on the Construction of a National Unified Market Logistics Channel” (PONE-D-23-19733). Those comments are very valuable and helpful for uw to revise and improve our work. We have revised the manuscript carefully on the basis of these comments and suggestions. The changes of our manuscript have been highlighted with track changes in color.

Our response are shown below for your considerations. We highly appreciate the valuable comments and suggestions. We hope the revised manuscript could eventually satisfy your requirements.

Sincerely yours,

Guangsheng Zhang

**Replies to Reviewer #1:**

**Reviewers' comments 1:**

1. What are the research objective? This should be clearly explained in introduction section.

**Response:**

We highly appreciate your valuable comments. To address this issue, We added the purpose of the research in introduction to clarify the main research motivation of this paper. At the same time, we also put forward the research purpose in abstract, so that readers can better understand the content of this paper.

**Reviewers' comments 2:**

2. Authors needs to discuss the logistics industry and game modeling at a broad level and cite the relevant articles. Following articles can be helpful for authors:

https://doi.org/10.1016/j.jclepro.2020.123127

https://doi.org/10.1108/IJRDM-03-2023-0177

**Response:**

We highly appreciate your valuable comments. To address this issue, after reading carefully the important literature recommended by the reviewers，the literature has been incorporated as a citation. At the same time, referring to the modification suggestions, we have added a description of the evolutionary game theory, which makes the logic of the paper clearer.

**Reviewers' comments 3:**

3. Citations are missing. Please recheck all the cited articles and ensure that all cited papers are listed in references.

**Response:**

We highly appreciate your valuable comments. To address this issue, we examined the full text of the paper carefully and reorganized the references.

**Reviewers' comments 4:**

4. Discussion section should be separate from conclusion section. Authors should include one "Discussion Section" and all the key findings should be presented there with the support of previously published literature.

**Response:**

We highly appreciate your valuable comments. To address this issue, we revised the content of conclusion and revelation section, added the previous key literature in conclusion, and more clearly explained the main theoretical contribution and academic value of this paper.

**Replies to Reviewer #2:**

**Reviewers' comments 1:**

ABSTRACT:

The abstract lacks contextual background and should provide a clear introduction to the significance of logistics channel construction in the national unified market. The study objectives should be expressed properly, and particular findings or conclusions should be given. For greater comprehension, the abstract's language and clarity should be enhanced.

**Response:**

We highly appreciate your valuable comments. To address this issue, we clearly introduce the significance of the logistics channel construction, and clarify the main research purpose of this paper in abstract. At the same time, we modify and comb the language description of the abstract, so that readers can better understand the content of this paper.

**Reviewers' comments 2:**

INTRODUCTION:

The introduction establishes a strong basis by highlighting the significance of logistics channel development in the national unified market. However, it would benefit from incorporating relevant citations, clearly stating the research gap and objectives, discussing the significance and practical implications of the research, and improving the overall structure and organization.

**Response:**

We highly appreciate your valuable comments. To address this issue, we clarify the important role of the government in the construction of logistics channels in introduction, and put forward the main research purpose and practical significance of this paper, which improves the readability and standardization of this paper.

**Reviewers' comments 3:**

LITERATURE REVIEW:

The literature review could be more organized and structured, grouping studies according to key findings or research focus. It should give a more critical review and synthesis of the previous literature, noting gaps or unsolved issues that the present study is attempting to address.

**Response:**

We highly appreciate your valuable comments. To address this issue, we have divided the relevant studies into two categories, one is the literature on the construction of logistics channels with government participation, and the other is the literature on the application of game theory in the coordination of logistics channel stakeholders. We point out the differences between this study and the current literature and illustrate the key issues that this paper seeks to address.

**Reviewers' comments 4:**

METHODS:

The methodology is well-structured, but further details on the specific simulation setup, assumptions, and criteria used for selecting optimal assignment parameters should be provided. The paper should also consider conducting robustness checks, sensitivity analyses, and discussing the limitations of the numerical experiments.

**Response:**

We highly appreciate your valuable comments. To address this issue, in the numerical experiment section, we illustrate the principle of setting basic parameters in this paper, point out the value and specific range of the model. At the same time, sensitivity analysis is used to clarify the influence mechanism of initial participation intention, positive cooperation benefit, cost proportion coefficient, penalty coefficient and construction cost under the two modes.

**Reviewers' comments 5:**

DISCUSSION AND CONCLUSION:

The conclusion effectively summarizes the research findings and offers valuable management implications. However, it could be strengthened by providing a brief reflection on the significance of the research in the broader context of logistics channel construction and the national unified market.

**Response:**

We highly appreciate your valuable comments. To address this issue, from a broader background, we propose that the logistics channel can promote the flow of commodity elements in a wider range in management enlightenment, which is conducive to the construction of a unified national market.

**Replies to Reviewer #3:**

**Reviewers' comments 1:**

Introduction: The abstract needs a clear motivation for the research and the significance of studying logistics channel construction in a national unified market. Providing a brief background on the importance of efficient logistics channels in supporting economic development and regional coordination would be beneficial.

**Response:**

We highly appreciate your valuable comments. To address this issue, in abstract, we clearly introduce the significance of logistics channel construction, clarify the main research purpose of this paper, and introduce the importance and significance of efficient logistics channels in supporting economic development and regional coordination.

**Reviewers' comments 2:**

Objectives and Research Questions: Clearly state the objectives and research questions addressed in the study. It will help readers understand the purpose and scope of the research more effectively.

**Response:**

We highly appreciate your valuable comments. To address this issue, we clearly illustrate the purpose and the research questions involved in introduction, which helps the reader to more effectively understand the purpose and scope of the research.

**Reviewers' comments 3:**

Methodology: Provide a concise overview of the evolutionary game theory approach used in the study, highlighting its advantages over traditional game theory in addressing logistics channel construction. Explain the rationale for assuming bounded rationality and how it impacts the model's outcomes.

**Response:**

We highly appreciate your valuable comments. To address this issue, we clearly explain the difference between the evolutionary game theory and the traditional game theory in introduction, and show that the evolutionary game theory can more truly reflect the diversity and complexity of the logistics channel actors.

**Reviewers' comments 4:**

Results and Discussion: Elaborate on the numerical and Matlab software simulations conducted to explore the impact of various factors on strategic decisions. Present the results clearly and structured, discussing the implications of these findings for logistics enterprises and government policymakers. Additionally, consider presenting any limitations of the simulations and possible areas for future research.

**Response:**

We highly appreciate your valuable comments. To address this issue, we propose model limitations and point out possible areas for future research directions.

**Reviewers' comments 5:**

Conclusion: Summarize the essential findings and their implications for logistics channel construction in a national unified market. Highlight the practical significance of the research and provide actionable recommendations for government and logistics enterprises to enhance the coordination and development of logistics channels.

**Response:**

We highly appreciate your valuable comments. To address this issue, in the part of management enlightenment, based on a broader background, we propose that the construction of logistics channels will help to strengthen the smooth connectivity of the domestic market, promote the active connection between the domestic market and the international market, and thus be conducive to the construction of a unified national market. Finally, we provide operational suggestions for the government and logistics enterprises to strengthen the coordination and development of logistics channels.

**Reviewers' comments 6:**

Language and Clarity: Ensure the abstract is written in clear and concise language, avoiding jargon or technical terms that may be unfamiliar to a broad audience. Proofread the abstract to eliminate any grammatical errors or awkward phrasing.

**Response:**

We highly appreciate your valuable comments. To address this issue, we reproofread the full-text language to make it more clear and concise. At the same time, we also proofread the abstract section again to avoid grammatical errors.
