# Supplementary material for: A study on the promotion effect of government guidance on the construction of a national unified market logistics channel
Source: PLoS One. 2023 Nov 13;18(11):e0293969. doi: 10.1371/journal.pone.0293969 (PMC10642817; doi:10.1371/journal.pone.0293969)
Supplement: Attachment — Submitted filename: Response to Reviewers v2.docx [file pone.0293969.s002.docx]

Dear Editors and Reviewers:

We appreciate Editor’s sincere letter and Reviewers' helpful comments concerning our manuscript entitled “A Study on the Promotion Effect of Government Guidance on the Construction of a National Unified Market Logistics Channel” (PONE-D-23-19733). Those comments are very valuable and helpful for revising and improving our paper. We have carefully revised the manuscript on the basis of the comments and suggestions. We highlight the changes in our manuscript with track changes in color.

**Replies to Reviewer #2:**

**Reviewers' comments 1:**

Abstract

1. "and to realize regional economic integration, it is of great value to the implementation of the national unified market strategy."

a. The phrase "it is of great value to the implementation" is a bit awkward. It could be rephrased for clarity, such as "it greatly contributes to the implementation."

2. "clarifies the effect of government participation and support policies by clarifying the role and functions of the government"

a. The repetition of "clarifying" is redundant. It can be improved by saying "by defining the role and functions of the government."

3. "We constructs an evolutionary game model among participating stakeholders, then studies the evolutionary stabiliy strategy"

a. "We constructs" should be corrected to "We construct." It's a subject-verb agreement issue.

b. "studies the evolutionary stabiliy strategy" should be corrected to "study the evolutionary stability strategy."

4. "influences each other, and government participation can change the effects of cooperative income,"

a. The subject-verb agreement is incorrect. It should be "influence each other" to match the plural subject.

5. "the government should provide reasonable guidance to prevent adverse selection of enterprises from hindering the healthy development of logistics channels."

a. The phrase "prevent adverse selection of enterprises from hindering" is somewhat complex. It could be simplified to improve readability, such as "to prevent enterprises from hindering."

**Response:**

We highly appreciate your valuable comments. To address this issue, we have polished the abstract on the basis of the modification requirements, which improved the readability and professionalism of abstract.

**Reviewers' comments 1:**

Introduction

The language used is generally clear and academic. However, there are some areas where sentence structure could be simplified for greater clarity and readability. For instance, in the sentence "differences in logistics channel participation behavior under the guidance of market mechanism and government are compared, and the difficulty of realizing and maintaining system stability in the two modes is analyzed," it might be clearer to split this into two sentences.

**Response:**

We highly appreciate your valuable comments. To address this issue, We simplified the sentence structure according to the modification requirements, “we compares the differences of the participation behavior of the logistics channel between market mechanism and government guidance, analyzed the difficulty of realizing and maintaining the system stability under these two modes. ”

In addition, we proofread the entire work, which further improved the overall quality of this paper.

Once again, we would like to stress that we highly appreciate the valuable comments and suggestions. We hope the revised manuscript could eventually satisfy your requirements.

Sincerely,

Guangsheng Zhang

Shandong Management University,

Jinan, Shandong, 250357, P R China.
